# De novo transcriptome sequencing of two cultivated jute species under salinity stress

**DOI:** 10.1371/journal.pone.0185863

**Published:** 2017-10-23

**Authors:** Zemao Yang, An Yan, Ruike Lu, Zhigang Dai, Qing Tang, Chaohua Cheng, Ying Xu, Jianguang Su

**Affiliations:** 1 Institute of Bast Fiber Crops, Chinese Academy of Agricultural Sciences/Key Laboratory of Stem-fiber Biomass and Engineering Microbiology, Ministry of Agriculture, Changsha, China; 2 Natural Sciences and Science Education, National Institute of Education, Nanyang Technological University, Singapore, Singapore; Jawaharlal Nehru University, INDIA

## Abstract

Soil salinity, a major environmental stress, reduces agricultural productivity by restricting plant development and growth. Jute (*Corchorus* spp.), a commercially important bast fiber crop, includes two commercially cultivated species, *Corchorus capsularis* and *Corchorus olitorius*. We conducted high-throughput transcriptome sequencing of 24 *C*. *capsularis* and *C*. *olitorius* samples under salt stress and found 127 common differentially expressed genes (DEGs); additionally, 4489 and 492 common DEGs were identified in the root and leaf tissues, respectively, of both *Corchorus* species. Further, 32, 196, and 11 common differentially expressed transcription factors (DTFs) were detected in the leaf, root, or both tissues, respectively. Several Gene Ontology (GO) terms were enriched in NY and YY. A Kyoto Encyclopedia of Genes and Genomes analysis revealed numerous DEGs in both species. Abscisic acid and cytokinin signal pathways enriched respectively about 20 DEGs in leaves and roots of both NY and YY. The Ca^2+^, mitogen-activated protein kinase signaling and oxidative phosphorylation pathways were also found to be related to the plant response to salt stress, as evidenced by the DEGs in the roots of both species. These results provide insight into salt stress response mechanisms in plants as well as a basis for future breeding of salt-tolerant cultivars.

## Introduction

Soil salinity is a major environmental stress imposed on plants that reduces agricultural productivity by restricting plant development and growth[1]. Salinity has primary effects including ion toxicity and osmotic stress as well as secondary effects such as oxidative stress[1]. Plants have a variety of salt tolerance mechanisms that depend on mitogen-activated protein kinase (MAPK/MPK) and hormone signaling as well as posttranslational modification of proteins.

Na^+^ influx into roots occurs via different transporters. Plants use the Na+/H+ salt overly sensitive (SOS)1 antiporter, high-efficiency potassium transporter (HKT), and the tonoplast-localized Na^+^, K^+^/H^+^ exchanger (NHX) for sodium transport and detoxification[2, 3]. Na^+^ influx triggers an increase in cytosolic Ca^2+^ level; this is sensed by SOS3, which activates the serine/threonine protein kinase SOS2. Activated SOS2 phosphorylates and activates SOS1[2], a plasma membrane Na+/H+ antiporter that plays an important role in maintaining a low concentration of Na^+^ in the cytoplasm of cortex cells by extruding Na^+^ into the soil and loading Na^+^ into the xylem for long-distance transport to leaves[4, 5]. HKT1 mediates the reverse flux and unloads Na^+^ from xylem vessels to prevent accumulation of Na^+^ in the transpirational stream.

Salt stress can lead to elevated levels of reactive oxygen species (ROS) such as superoxide anion and hydrogen peroxide (H_2_O_2_) that are toxic and can cause oxidative damage to proteins, DNA, and lipids in the cell membrane[6]. ROS are scavenged by antioxidant metabolites (e.g., ascorbate and glutathione) and by ROS-detoxifying enzymes (e.g., superoxide dismutase and ascorbate peroxidase). Despite their toxicity to cells, ROS also function as signal transduction molecules that mediate responses to stress[7] by activating various MAPK signaling cascades, including MAPK kinase kinase 1, MPK4, and MPK6[8, 9].

Hormone signaling is important for mediating salt stress responses in plants[2]. For example, when abscisic acid (ABA) accumulates and binds to the pyrabactin resistance/Pyr1-like (PYL)/regulatory components of ABA receptor, the resultant conformational change leads to interaction with protein phosphatase (PP)2C and the formation of the ternary ABA-ABAR-PP2C complex. This in turn relieves inhibition of sucrose non-fermenting-1-related protein kinase (SnRK)2 via PP2C suppression. SnRK2 then phosphorylates and activates ABA response element-binding factor (ABF/AREB)/ABA-INSENSITIVE 5 transcription factors (TFs), resulting in ABA-dependent gene expression. During the abiotic stress response, ABA signaling activates the MAPK cascade, which regulates ABA effector proteins[10]. ABA can also induce H_2_O_2_ generation via phosphorylation of the plasma membrane NADPH oxidase respiratory burst oxidase homolog protein F. H_2_O_2_ can then mediate various ABA responses by modulating Ca^2+^ signaling[1].

Jute (*Corchorus* spp.) is one of the most commercially important bast fiber crops in the world as it provides biodegradable and renewable lignocellulose fiber. *Corchorus capsularis* and *Corchorus olitorius* are two commercially cultivated species of jute[11]. The plant is mainly distributed in China, India, Bangladesh, and east-central Africa[12] but global demand has been increasing due to its broad-spectrum applications and environmentally friendly characteristics. Various studies have investigated jute salt tolerance[13]. Naik et al. [14] studied a few of *Corchorus capsularis* accessions under differential salt concentration, and the results showed that some varieties could be grown at 160 mM NaCl concentration. Taneenah et al. studied the germination of *Corchorus olitorius* L underlying the Dead Sea water, sea water and water with differential NaCl concentration, and the results indicated that *Corchorus olitorius* L. showed high tolerance to Dead Sea water, sea water and NaCl up to 6‰[15]. So far, which one is comparatively more tolerant between *Corchorus capsularis* and *Corchorus olitorius* has not been determined, and it maybe depend on which accessions or varieties[16]. However, the underlying mechanism has not been reported at the molecular level. To this end, the present study investigated the gene expression profiles of *C*. *capsularis* and *C*. *olitorius* under salt stress by high-throughput transcriptome sequencing. Our findings provide some insight into the mechanisms of salt tolerance in plants.

## Materials and methods

### Plant materials and salt stress treatment

Yueyuan NO.5, an important cultivar in China of *C*. *capsularis* L. (named YY) and NY/253C, a accession of *C*. *olitorius* L. (named NY) originated from China were hydroponically cultivated at 25°C–28°C in Yoshida nutrient solution in a greenhouse. And the salt tolerance of YY was only slightly better than that of NY. At the nine-leaf stage, six uniform seedlings of each species were selected; three were treated with 250 mM NaCl and the other three served as controls. After 12 h, the roots and leaves were collected for RNA extraction.

### RNA isolation and transcriptome sequencing

Total RNA was extracted from tissue samples using TRIzol reagent (Invitrogen, Carlsbad, CA, US) according to the manufacturer’s protocol. RNA degradation and contamination was monitored in 1% agarose gels. RNA purity, integrity and the concentration was measured with a NanoPhotometer spectrophotometer (Implen, Westlake Village, CA, USA), RNA Nano 6000 Assay kit with the Bioanalyzer 2100 system (Agilent Technologies, Santa Clara, CA, USA), and Qubit RNA Assay kit with the Qubit 2.0 flurometer (Life Technologies, Carlsbad, CA, USA). A total of 24 RNA sequencing libraries (NYCR, NY control root; NYCL, NY control leaf; NYSR, NY salt-stressed root; NYSL, NY salt-stressed leaf; YYCR, YY control root; YYCL, YY control leaf; YYSR, YY salt-stressed root; YYSL, YY salt-stressed leaf. three biological repetition for above each sample) were generated using the NEBNext Ultra RNA Library Prep kit for Illumina (New England Biolabs, Ipswich, MA, USA) according to the manufacturers’ instructions. The quality of the libraries was assessed with the Agilent Bioanalyzer 2100 system. Libraries were sequenced on an Illumina HiSeq 4000 platform (San Diego, CA, USA) and paired-end reads were generated for transcriptome sequencing.

### Transcriptome data analysis and annotation

Quality control of raw data was carried out with Perl scripts developed in house by removing reads containing adapter sequences, poly-N, and low-quality reads. Clean reads were assembled into a transcriptome using Trinity[17] with default settings for all parameters and mapped to transcripts; those with less than 5× coverage were removed. The transcripts which would be used as reference transcripts for analysis of DEGs were further assembled into non-redundant unigenes. Gene function was annotated using the following databases: National Center for Biotechnology Information (NCBI) non-redundant protein sequences (Nr); NCBI non-redundant nucleotide sequences (Nt); Protein Family (Pfam); Clusters of Orthologous Groups of Proteins (KOG); Swiss-Prot, Kyoto Encyclopedia of Genes and Genomes (KEGG) Ortholog (KO) database; and Gene Ontology (GO) using the Basic Local Alignment Search Tool with an E-value threshold of 10−5[18]

### Analysis of differentially expressed genes (DEGs)

Gene expression levels for each sample were estimated using RSEM[19]. Clean data from each sample were respectively mapped back onto the assembled transcripts, and the mapping results for each gene were used for differential expression analysis of unigenes. Differential expression analysis of the two conditions was carried out using the DESeq R package (1.10.1)[20]. Genes with P_adjusted_ < 0.05 assigned by DESeq were considered as differentially expressed. GO enrichment and KEGG pathways analysis of DEGs were implemented with the GOseq R package[21] and KOBAS[22] software.

### Quantitative real-time (qRT)-PCR analysis

Eight DEGs randomly selected from the RNA-seq results and eight DEGs selected from the list of some differentially expressed genes (DEGs) playing important roles in salinity stress were analyzed by qRT-PCR to validate RNA-seq results in the same samples used for RNA-seq with two independent biological and three technological replicates. The jute *elongation factor-alpha* (*ELF*) gene was used as the endogenous control[23]. Primer sequences for amplifying DEGs and *ELF* were listed in S1 File. For qRT-PCR, total RNA was reverse transcribed into first-strand cDNA using the M-MuLV Reverse Transcriptase kit (Fermentas, Burlington, ON, Canada) according to the manufacturer’s instructions. The reaction was performed using the FastStart Universal SYBR Green Master kit (Roche Diagnostics, Basel, Switzerland) according to the manufacturer’s instructions in an optical 384-well plate on a GeneAmp PCR System 9700 (Applied Biosystems, Foster City, CA, USA). The cycle threshold method was used to calculate relative expression levels[24].

## Results

### Transcriptome assembly and functional annotation

A total of 24 NY and YY samples under salt stress and control conditions were used for Illumina paired-end transcriptome sequencing and analysis. We obtained 1,234,376,376 raw reads (Table 1). After quality control, 1,200,159,212 clean reads (97.23% of raw reads) were obtained with a high base quality score (average Q20 = 97.51%), accounting for approximately 180.06 Gb of sequencing data. A total of 161,760 transcripts were assembled with lengths varying from 201 to 15,953 bp and an average length of 1578 bp (Table 2). Transcripts were assembled into 72,278 non-redundant unigenes with an average length of 1240 bp. All sequencing data were deposited into the NCBI SRA database under accession numbers SRP116874 (included sequence data of 12 NY samples) and SRP119015 (included sequence data of 12 YY samples and unigenes annotation files).

**Table 1:** Details of the raw and clean data of 24 NY and YY sample transcriptomes.

Genotypes	Tissues	Raw reads		Clean reads		Clean bases(Gb)(GC%)		Raw reads avg Q20
		Control	Salinity	Control	Salinity	Control	Salinity	(%)
NY	Leaf	154,357,990	157,905,474	149,840,624	151,807,922	22.48(45.27%)	22.78(45.12%)	97.51
	Root	170,983,540	150,144,930	165,732,832	145,221,480	24.86(44.06%)	21.78(44.05%)	
YY	Leaf	160,566,334	148,384,870	157,631,384	145,816,236	23.64(43.61%)	21.88(43.54%)	
	Root	156,290,594	135,742,644	151,681,828	132,426,906	22.76(43.47%)	19.88(43.34%)	
Total		1,234,376,376		1,200,159,212 (97.23%)		180.06		

**Table 2:** Assembly output statistics of all clean data using the Trinity assembler.

Parameters	Value
Number of unigenes	72,278
Number of Transcripts	161,760
Average unigenes length (bp)	1,240
Average Transcripts length (bp)	1,578
Min length unigenes length (bp)	201
Min length Transcripts length (bp)	201
Max length unigenes length (bp)	15,953
Max length Transcripts length (bp)	15,953

**Fig 1:**
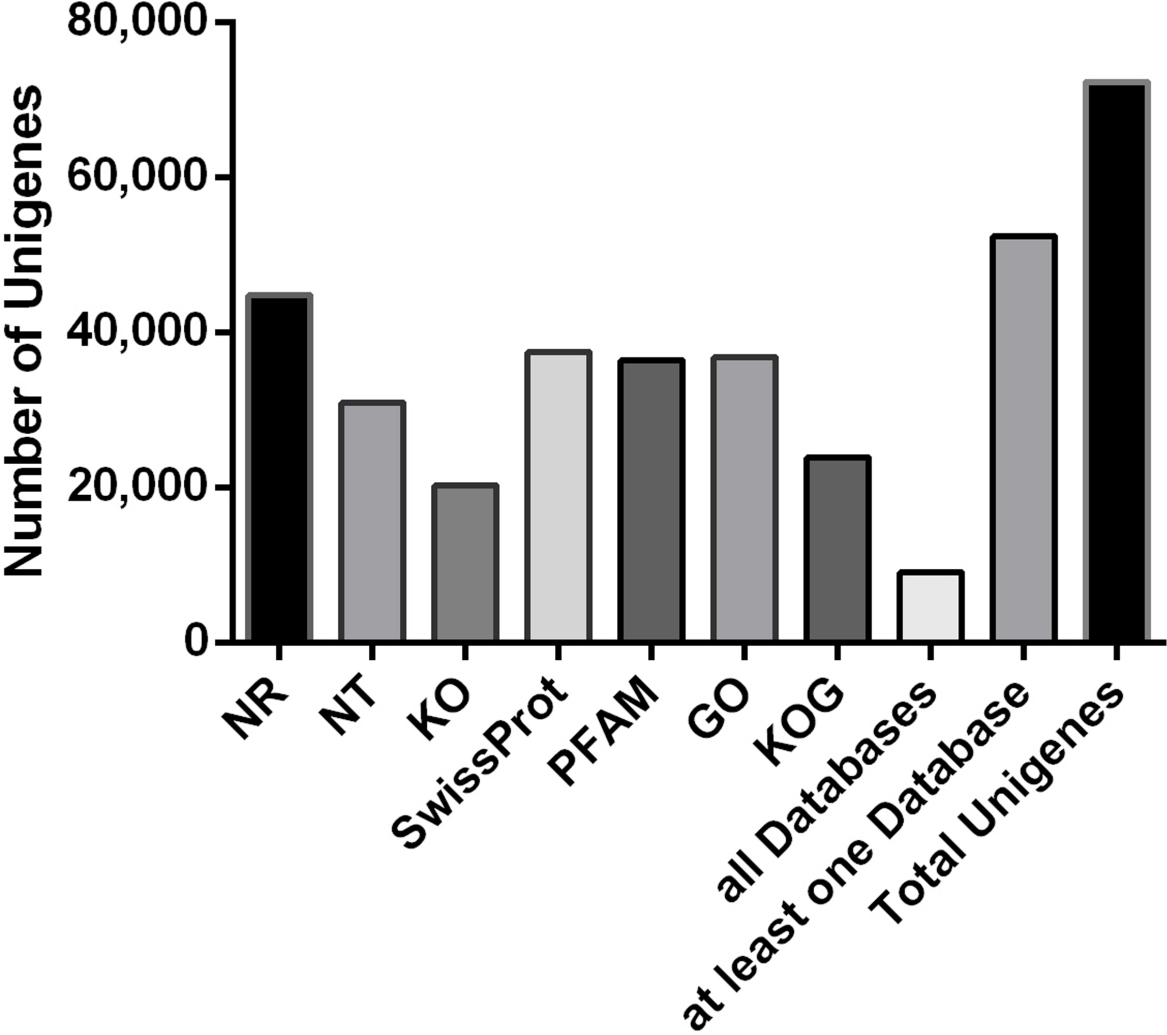
Unigenes annotated success across multiple databases.

In total, 9108 (12.6%) unigenes were found to have homologs in the seven databases (Fig 1), with 52,417 (72.52%) annotated in at least one database. The largest number of unigenes (44,764, 61.93%) had hits to protein sequences in Nr, while only 20,283 (28.06%) were mapped in KO.

### Differential gene expression in response to salt treatment

We analyzed genes that were differentially expressed in various *Corchorus* tissues in response to salt stress. In general, there were more DEGs in roots than in leaves in the two species. There were 1932 (792/1140) and 9608 (1904/7704) (S2 File, Table 3) DEGs (up-/downregulated) in NY and 3089 (1799/1290) (S2 File, Table 3) and 15,099 (6401/8698) (S2 File, Table 3) DEGs (up-/downregulated) in YY in the leaf and root tissues, respectively. A comparative analysis revealed that 4489 (948/3294) (S3 File, Table 3) and 492 (181/131) (S4 File, Table 3) root and leaf DEGs (upregulated/downregulated), respectively, were to both species, while 127 (107/12) (S5 File, Table 3) DEGs (upregulated/downregulated) were present in both tissues of both species.

**Table 3:** Statistics of differentially expressed genes (DEGs) in multiple NY and YY comparison groups under salt stress and control conditions.

Comparison groups	No. DEG	Upregulated	Downregulated
NYSL_vs_NYCL	1932	792	1140
NYSR_vs_NYCR	9608	1904	7704
YYSL_vs_YYCL	3089	1799	1290
YYSR_vs_YYCR	15099	6401	8698
NYSR_vs_NYCR & YYSR_vs_YYCR	4489	948	3294
NYSL_vs_NYCL & YYSL_vs_YYCL	492	181	131
NYSL_vs_NYCL&YYSL_vs_YYCL&NYSR_vs_NYCR&YYSR_vs_YYCR	127	107	12

NYSR (NY salt-stressed root); NYCR (NY control root); NYSL (NY salt-stressed leaf); NYCL (NY control leaf); YYSR (YY salt-stressed root); YYCR (YY control root); YYSL (YY salt-stressed leaf); TCCL (TC control leaf)

### Differentially expressed (D)TFs

**Fig 2:**
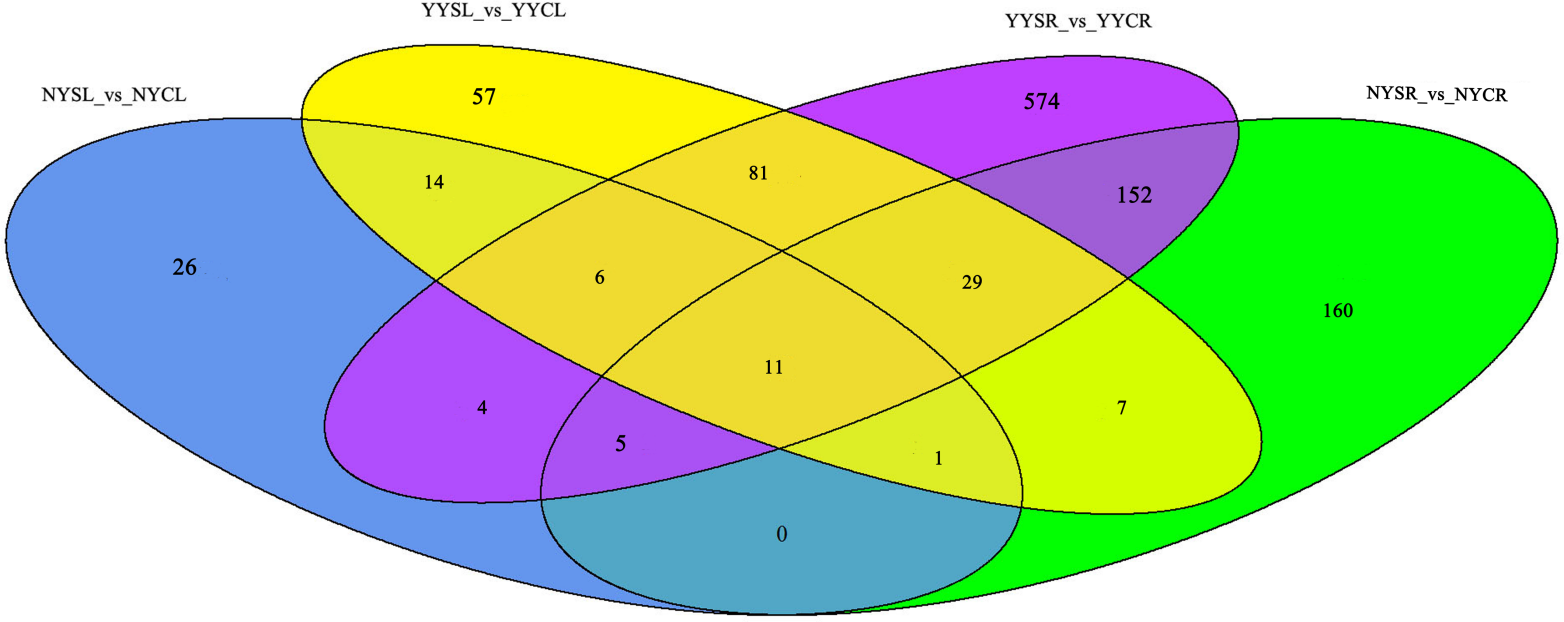
DTFs in the roots and leaves of *C*. *capsularis* and *C*. *olitorius* cultivated under high salt conditions. DTFs, Differentially expressed (D)TFs; NYCL, NY control leaf; NYCR, NY control root; NYSL, NY salt-stressed leaf; NYSR, NY salt-stressed root; YYCL, YY control leaf; YYCR, YY control root; YYSL, YY salt-stressed leaf; YYSR, YY salt-stressed root.

In the study, a total of 2303 TFs were discovered (S6 File). There were more DTFs in YY than in NY (206/862 and 67/365 DTFs in leaves/roots of YY and NY, respectively) (Fig 2). In the leaves, there were 32 DTFs common to the two species (S7 File and Fig 2). These DTFs belonged to 17 TF families, with homeobox (HB) and heat shock factor (HSF) being the most highly represented. In the roots, there were 196 DTFs (S8 File and Fig 2) from 43 TF families, of which six had more than 10 unigenes including myeloblastosis (MYB) (25), WRKY (12), CCAAT (12), Apetala (AP)2/ethylene response element binding protein (EREBP) (11), Orphans (10), and forkhead-associated domain (FHA) (10). There were 11 DTFs (S9 File and Fig 2) common to both tissues and both species, including four HSF, three HB, and one each of NAM/ATAF/CUC (NAC), basic leucine zipper (bZIP), CCAAT, and tafazin.

### GO and KEGG analyses of DEG function

**Fig 3:**
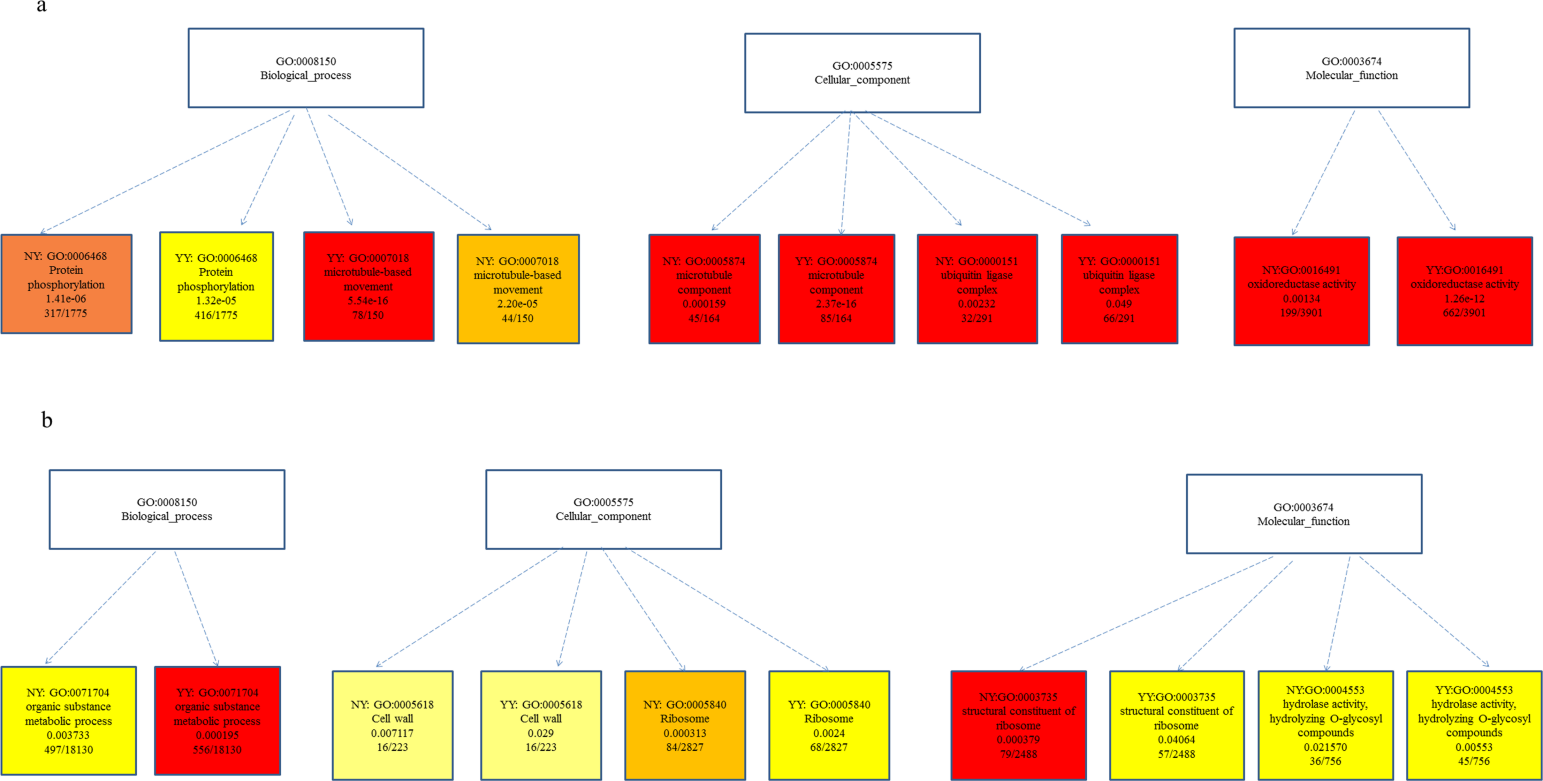
Differentially expressed genes (DEGs) induced by salt stress in both NY and YY root (a) and leaf (b) tissues of *Corchorus* according to GO term annotation analysis.

To gain insight into the functions of DEGs induced by salt stress in root and leaf tissues of *Corchorus*, we analyzed GO term annotations. In the roots, massive downregulated unigenes were enriched in the GO terms ‘protein phosphorylation’ (GO:0006468), ‘microtubule-based movement’ (GO:0007018), and ‘microtubule component’ (GO:0005874), whereas numerous upregulated unigenes were enriched in ‘ubiquitin ligase complex’ (GO:0000151) and ‘oxidoreductase activity’ (GO:0016491) (Fig 3A). In leaves, the GO terms ‘organic substance metabolic process’ (GO:0071704), ‘cell wall’ (GO:0005618), ‘ribosome’ (GO:0005840), ‘structural constituent of ribosome’ (GO:0003735), and ‘hydrolase activity/hydrolyzing *O*-glycosyl compounds’ (GO:0004553) were significantly enriched in both NY and YY (Fig 3B).

In order to identify pathways involved in the salt stress response, we carried out a KEGG pathways analysis of DEGs and found that pathways associated with metabolism and signaling were highly represented. There were 13 pathways enriched in both tissues in both species (Table 4) such as those related to plant hormone signal transduction and the peroxisome; and six pathways (Table 4) were enriched in roots in both NY and YY, including MAPK and Ca^2+^ signaling. On the other hand, methane metabolism and cutin, suberin, and wax biosynthesis were only enriched in leaves of both species, although methane metabolism accounted for 10 and nine DEGs in NYL and YYL, respectively, and cutin, suberin, and wax biosynthesis accounted for four DEGs in the leaves of NY and YY (here, we only list the number of DEGs within enriched pathways in root and leaf of NY or YY in Table 4, while a few of DEGs (or no DEGs) without obviously enrichment in root and leaf of NY or YY were not showed (such as TCA cycle, only in root enrichment). Of the 127 DEGs common to both tissues and species, 13 were enriched in plant hormone signal transduction.

**Table 4:** The differentially expressed genes (DEGs) number and pathways associated with metabolism and signaling highly represented under salt stress.

Pathway ID	The number of differentially expressed genes				The number of backgroud genes	Pathway terms
	NYSL vs. NYCL	YYSL vs. YYCL	NYSR vs. NYCR	YYSR vs. YYCR		
ko01210	9	11	23	42	164	2-Oxocarboxylic acid metabolism
ko00520	19	21	29	60	213	Amino sugar and nucleotide sugar metabolism
ko00330	11	14	34	51	179	Arginine and proline metabolism
ko01230	50	39	67	130	570	Biosynthesis of amino acids
ko00710	18	19	31	42	236	Carbon fixation in photosynthetic organisms
ko01200	45	39	83	136	676	Carbon metabolism
ko00260	16	15	31	44	168	Glycine, serine and threonine metabolism
ko00040	10	13	24	45	156	Pentose and glucuronate interconversions
ko00030	8	9	17	37	135	Pentose phosphate pathway
ko00360	15	22	33	92	175	Phenylalanine metabolism
ko00940	24	33	40	125	240	Phenylpropanoid biosynthesis
ko04075	28	57	49	125	288	Plant hormone signal transduction
ko04146	14	15	63	68	262	Peroxisome
ko00020	-	-	44	62	267	Citrate cycle (TCA cycle)
ko00190	-	-	59	57	738	Oxidative phosphorylation
ko04010	-	-	47	49	292	MAPK signaling pathway
ko04020	-	-	45	38	213	Calcium signaling pathway
ko04120	-	-	43	50	322	Ubiquitin mediated proteolysis
ko00250	-	-	14	25	98	Alanine, aspartate and glutamate metabolism
ko00680	10	9	-	-	115	Methane metabolism
ko00073	4	4	-	-	26	Cutin, suberine and wax biosynthesis

### Some DEGs playing important roles in salinity stress

Here, numerous DEGs that played important roles in salinity stress were discovered. In Table 5, we list DEGs encoding HKT1, CBL-interacting protein kinases (CIPK), late embryogenesis abundant protein gene (LAE) etc. In the DEGs, the most of DEGs were the unigenes encoding CIPK (27), and 17 were up-regulated, following by the DEGs encoding MKK (9) with 6 down-regulated, 2 up-regulated and one up and down-regulated in NY or YY. The two DEGs (c122990_g1 and c122990_g2) were both up-regulated in the two tissues of both species. The two LAE DEGs and two SOS1 DEGs were all up-regulated in roots of YY. And one HKT1 was upregulated only in NY root.

**Table 5:** the list of some differentially expressed genes (DEGs) playing important roles in salinity stress.

Gene ID	treatment vs. contral	Log2(FC)	q_value	code protein	gene_ length	function
c127423_g2	NYSR vs. NYCR	4.879	2.20E-02	HKT1	1983	transmembrane transport//cation transport
c108681_g1	YYSL vs. YYCL	2.927	1.70E-02	CIPK6	771	signal transduction//protein phosphorylation
c119693_g1	NYSR vs. NYCR	-4.625	8.15E-03	CIPK23	1762	protein phosphorylation
c120042_g2	YYSL vs. YYCL	4.023	1.87E-03	CIPK6	1880	protein phosphorylation//signal transduction
c122990_g1	NYSL vs. NYCL	1.752	2.91E-03	CIPK25	812	protein phosphorylation
c122990_g1	NYSR vs. NYCR	2.197	1.19E-08	CIPK25	812	protein phosphorylation
c122990_g1	YYSL vs. YYCL	1.874	1.00E-10	CIPK25	812	protein phosphorylation
c122990_g1	YYSR vs. YYCR	3.238	1.38E-84	CIPK25	812	protein phosphorylation
c122990_g2	NYSL vs. NYCL	1.802	5.54E-04	CIPK25	1877	signal transduction//protein phosphorylation
c122990_g2	NYSR vs. NYCR	2.079	1.39E-08	CIPK25	1877	signal transduction//protein phosphorylation
c122990_g2	YYSL vs. YYCL	1.850	1.49E-11	CIPK25	1877	signal transduction//protein phosphorylation
c122990_g2	YYSR vs. YYCR	3.182	4.45E-51	CIPK25	1877	signal transduction//protein phosphorylation
c125842_g1	NYSR vs. NYCR	1.844	4.46E-04	CIPK10	2412	protein phosphorylation//signal transduction
c125842_g1	YYSL vs. YYCL	0.777	3.18E-02	CIPK10	2412	protein phosphorylation//signal transduction
c125842_g1	YYSR vs. YYCR	1.939	2.10E-33	CIPK10	2412	protein phosphorylation//signal transduction
c126096_g1	YYSR vs. YYCR	-1.437	6.13E-18	CIPK7	1840	protein phosphorylation//signal transduction
c127863_g1	NYSR vs. NYCR	2.126	1.56E-06	CIPK2	3180	signal transduction//protein phosphorylation
c127863_g1	YYSL vs. YYCL	0.720	2.63E-02	CIPK2	3180	signal transduction//protein phosphorylation
c127863_g1	YYSR vs. YYCR	2.506	2.17E-35	CIPK2	3180	signal transduction//protein phosphorylation
c128981_g1	YYSL vs. YYCL	2.357	7.29E-08	CIPK25	2389	signal transduction//protein phosphorylation
c128981_g1	YYSR vs. YYCR	0.514	3.68E-03	CIPK25	2389	signal transduction//protein phosphorylation
c129602_g4	YYSR vs. YYCR	1.752	1.16E-31	CIPK6	1063	protein phosphorylation
c129602_g5	YYSR vs. YYCR	1.870	2.16E-36	CIPK6	922	protein phosphorylation//signal transduction
c130525_g2	NYSR vs. NYCR	1.148	2.41E-03	CIPK10	4968	protein phosphorylation//signal transduction
c130525_g2	YYSL vs. YYCL	0.945	1.72E-03	CIPK10	4968	protein phosphorylation//signal transduction
c130525_g2	YYSR vs. YYCR	0.782	1.46E-06	CIPK10	4968	protein phosphorylation//signal transduction
c131066_g1	NYSR vs. NYCR	2.018	7.03E-06	CIPK8	2721	signal transduction//protein phosphorylation
c131066_g1	YYSR vs. YYCR	1.402	2.44E-17	CIPK8	2721	signal transduction//protein phosphorylation
c132458_g2	YYSL vs. YYCL	1.034	1.31E-02	CIPK9	4385	signal transduction//protein phosphorylation
c132458_g2	YYSR vs. YYCR	0.889	1.69E-03	CIPK9	4385	signal transduction//protein phosphorylation
c133247_g1	NYSR vs. NYCR	1.526	6.50E-04	CIPK9	2301	protein phosphorylation
c133247_g1	YYSR vs. YYCR	2.933	9.96E-05	CIPK9	2301	protein phosphorylation
c133300_g1	NYSR vs. NYCR	3.610	7.49E-13	CIPK21	2694	protein phosphorylation//signal transduction
c133300_g1	YYSR vs. YYCR	1.167	3.19E-06	CIPK21	2694	protein phosphorylation//signal transduction
c133961_g3	NYSR vs. NYCR	1.918	4.84E-03	CIPK12	1865	protein phosphorylation//signal transduction
c133961_g3	YYSR vs. YYCR	1.073	1.15E-09	CIPK12	1865	protein phosphorylation//signal transduction
c134486_g1	YYSR vs. YYCR	0.946	2.35E-05	CIPK8	3079	protein phosphorylation//signal transduction
c135536_g3	YYSR vs. YYCR	-0.956	2.59E-10	CIPK32	1922	signal transduction//protein phosphorylation
c135536_g4	YYSR vs. YYCR	-1.240	3.51E-03	CIPK32	1084	signal transduction
c1961_g1	NYSR vs. NYCR	-7.549	3.43E-09	CIPK23	1477	protein phosphorylation//defense response to bacterium
c1961_g1	YYSR vs. YYCR	-5.827	4.89E-05	CIPK23	1477	protein phosphorylation//defense response to bacterium
c246105_g1	NYSR vs. NYCR	-4.464	6.58E-05	CIPK10	1435	protein phosphorylation
c248820_g1	NYSR vs. NYCR	-7.544	7.33E-09	CIPK30	1480	protein phosphorylation
c248820_g1	YYSR vs. YYCR	-5.389	2.20E-04	CIPK30	1480	protein phosphorylation
c253056_g1	NYSR vs. NYCR	-3.329	3.06E-02	CIPK11	1378	—
c68433_g1	NYSR vs. NYCR	-6.042	6.32E-09	CIPK28	1701	protein phosphorylation
c68433_g1	YYSR vs. YYCR	-4.270	2.94E-04	CIPK28	1701	protein phosphorylation
c72352_g1	NYSR vs. NYCR	-3.222	4.25E-02	CIPK30	891	protein phosphorylation
c90168_g1	NYSL vs. NYCL	5.569	9.11E-05	CIPK13	1424	protein phosphorylation
c129602_g4	YYSR vs. YYCR	1.752	1.16E-31	CIPK6	1063	protein phosphorylation
c111482_g1	YYSR vs. YYCR	1.401	1.70E-03	LEA	832	response to stress//response to water stimulus
c135934_g2	YYSL vs. YYCL	3.109	4.97E-07	LEA	1218	response to stress
c105006_g2	NYSR vs. NYCR	-3.547	3.62E-02	MKK5	1081	protein phosphorylation
c122526_g1	YYSR vs. YYCR	-1.317	8.13E-06	MKK6	1589	protein phosphorylation
c124453_g1	NYSR vs. NYCR	2.407	5.36E-06	MKK1	1461	protein phosphorylation
c124453_g1	YYSL vs. YYCL	-0.738	2.39E-02	MKK1	1461	protein phosphorylation
c124453_g1	YYSR vs. YYCR	3.907	5.36E-06	MKK1	1461	protein phosphorylation
c126229_g1	NYSR vs. NYCR	2.013	1.44E-07	MKK13	1486	protein phosphorylation//photosynthesis
c126229_g1	YYSR vs. YYCR	0.602	2.14E-02	MKK13	1486	protein phosphorylation//photosynthesis
c132388_g1	YYSR vs. YYCR	-0.419	2.31E-02	MKK5	3164	protein phosphorylation
c132606_g1	YYSR vs. YYCR	3.298	4.40E-51	MKK18	1537	thiamine biosynthetic process//protein phosphorylation
c207571_g1	NYSR vs. NYCR	-3.932	2.15E-03	MKK5	2599	protein phosphorylation//oxidation-reduction process
c207571_g1	YYSR vs. YYCR	-2.974	4.99E-02	MKK5	2599	protein phosphorylation//oxidation-reduction process
c226247_g1	NYSR vs. NYCR	-3.518	1.49E-02	MKK1	1057	protein phosphorylation
c81745_g2	NYSR vs. NYCR	-4.174	6.90E-03	MKK9	1669	protein phosphorylation
c121728_g1	YYSR vs. YYCR	-6.603	7.38E-22	NHX3	1516	cation transport//regulation of pH
c121728_g2	YYSL vs. YYCL	1.810	2.12E-02	NHX3	980	cation transport//regulation of pH
c181442_g1	NYSR vs. NYCR	-3.497	3.07E-02	NHX1	1290	cation transport//regulation of pH
c135331_g1	YYSR vs. YYCR	0.548	1.30E-03	SOS1	4448	transmembrane transport//regulation of pH
c135331_g1	YYSR vs. YYCR	0.548	1.30E-03	SOS1	4448	transmembrane transport//regulation of pH

### Differential expression of plant hormone signal transduction genes

**Fig 4:**
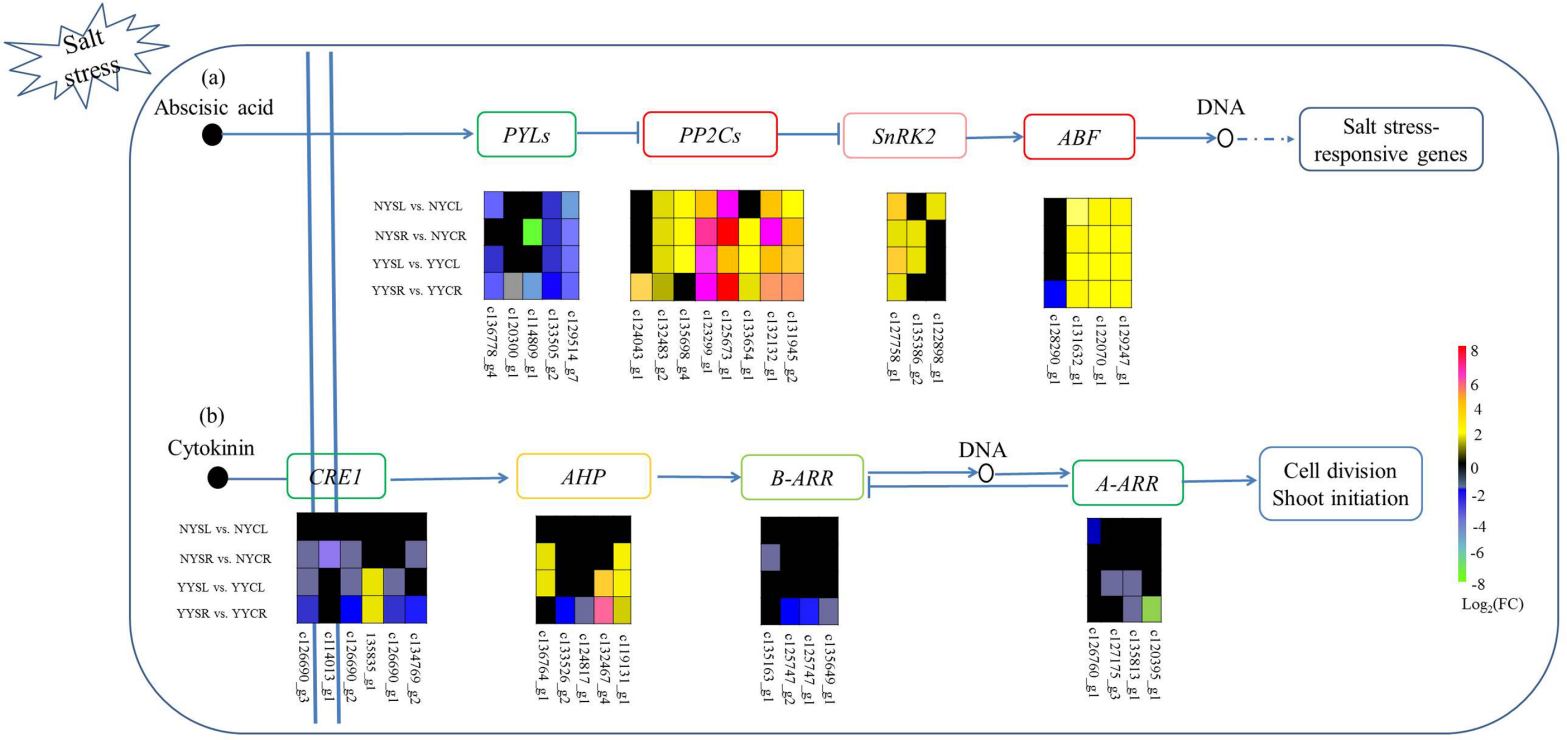
Differentially expressed genes (DEGs) belonging to the plant hormone pathway in root and leaf tissues of *C*. *capsularis* and *C*. *olitorius* cultivated under high salt conditions. (a) ABA and (b) cytokinin signaling pathways are shown. NYCR, NY control root; NYCL, NY control leaf; NYSR, NY salt-stressed root; NYSL, NY salt-stressed leaf; YYCR, YY control root; YYCL, YY control leaf; YYSR, YY salt-stressed root; YYSL, YY salt-stressed leaf.

Hormones play an important role in the response to different environmental stressors (e.g., drought and salt stress) in plants. In this study, we identified numerous DEGs associated with plant hormone (ABA, auxin, cytokinin, gibberellin, ethylene, jasmonic acid, and salicylic acid) signaling in leaves and roots of both NY and YY, with ABA and cytokinin signaling being the most highly represented. In the ABA pathway, two genes encoding PYL were downregulated in all tissues and species whereas three other genes were downregulated in a subset of tissues and in one of the species. Five, one, and three genes encoding PP2C, SnRK2, and ABF were upregulated in all tissues and both species, respectively; and five genes encoding the three proteins were upregulated while one was downregulated in subset of tissues and in one of the species (Fig 4A). In the cytokinin signaling pathway, 13 genes encoding cytokinin response (CRE)1 (5), B-APR (4), and A-type *Arabidopsis* response regulator (A-ARR) (4) were downregulated in at least one of four groups under salt treatment as compared to the control condition. Most genes encoding *Arabidopsis* histidine-containing phosphotransmitter (AHP) were upregulated in NYR, YYL, and YYR (Fig 4B).

### Pathways represented only in the roots of *Corchorus*

**Fig 5:**
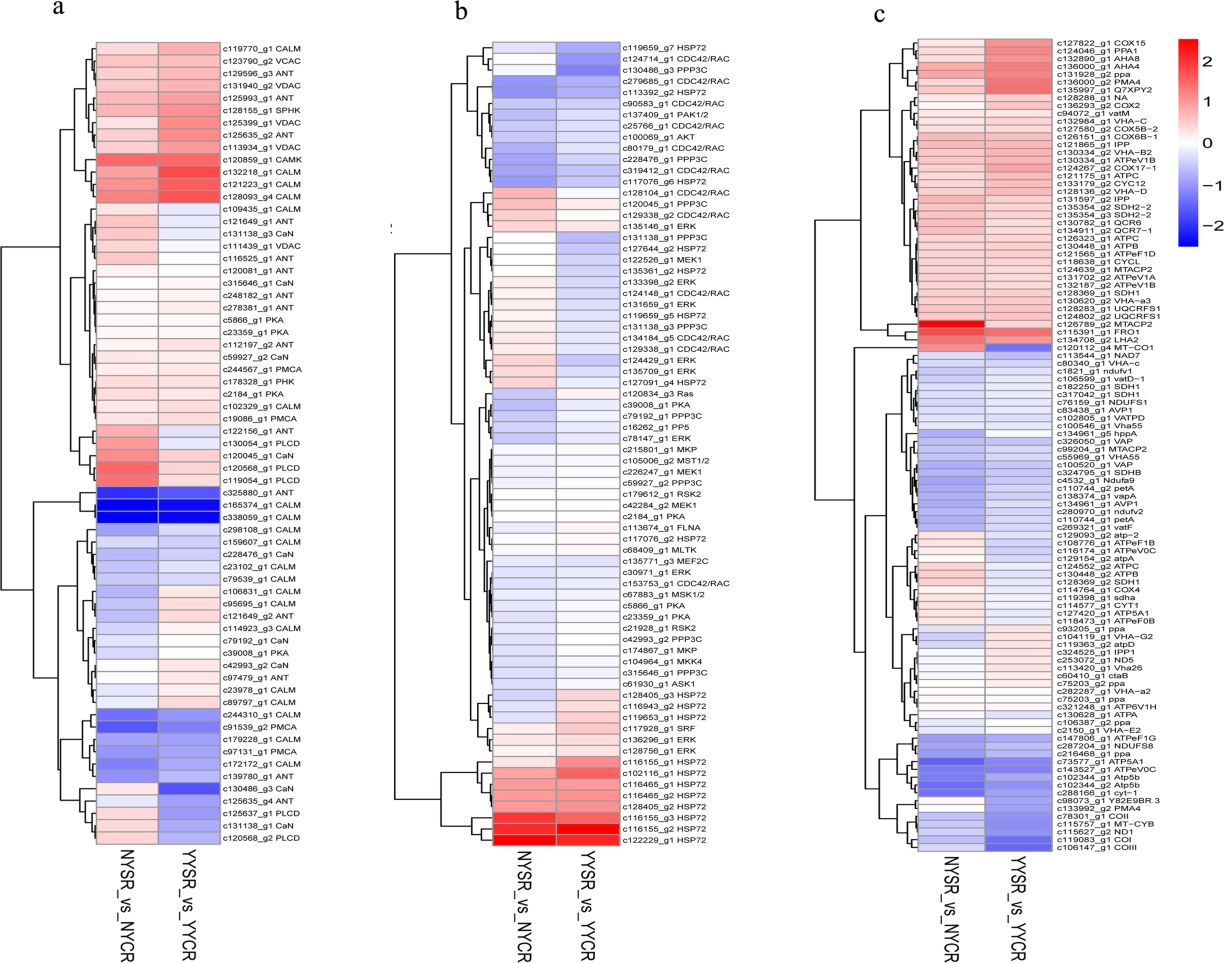
Differentially expressed genes (DEGs) belonging to the following signaling pathways in root tissue of *C*. *capsularis* and *C*. *olitorius* cultivated under high salt conditions. (a) Ca2+ signaling pathway; (b) MAPK signaling; and (c) oxidative phosphorylation pathways are shown. NYCR, NY control root; NYSR, NY salt-stressed root; YYCR, YY control root; YYSR, YY salt-stressed root.

The Ca^2+^, MAPK signaling and oxidative phosphorylation pathways play major roles in the plant response to salt stress. Numerous DEGs identified in our study were associated with these pathways. Components of the Ca^2+^ signaling pathway included calmodulin (CaM)/CaM-like (CML) proteins and Ca^2+^-dependent protein kinase (CAMK) (Fig 5A). A total of 20 genes encoding CML (4/16 up-/downregulated) were found in YY or NY roots, with most exhibiting similar expression in the two species. One upregulated gene encoding CAMK was common to both YY and NY roots. In addition, nine DEGs encoding serine/threonine-protein phosphatase 2B catalytic subunit, four encoding Ca^2+^-transporting ATPase, and four encoding protein kinase (PK)A were detected in YY or NY roots. In the MAPK pathway, DEGs encoding HSP72 (n = 19), Ras-related C3 botulinum toxin substrate 1 (n = 12), MAPK1/3 (n = 9), and PP3C (n = 8) were detected in YY and NY roots (Fig 5B). In the oxidative phosphorylation pathway, 19 DEGs encoding H^+^-transporting ATPase subunits (V and F types) and six encoding cytochrome c oxidase subunit were identified (Fig 5C). The expression profiles of DEGs in these three pathways were similar in the roots of the two species.

### Validation of DEGs

**Fig 6:**
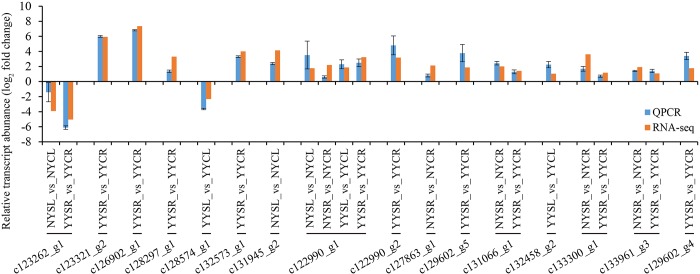
qRT-PCR validation of sixteen DEGs detected by RNA-seq. Data represent log2 (fold changes) of the relative transcript abundance of each DEG in NYSR (NY salt-stressed root) vs. NYCR (NY control root); NYSL (NY salt-stressed leaf) vs. NYCL (NY control leaf); YYSR (YY salt-stressed root) vs. YYCR (YY control root); and YYSL (YY salt-stressed leaf) vs. YYCL (YY control leaf). Red and blue represent expression levels determined by RNA-seq and qRT-PCR, respectively.

In order to validate the RNA-seq results, we carried out qRT-PCR analysis for eight randomly selected DEGs and eight DEGs selected from Table 5 in YY and NY under salt stress. All of the DEGs showed differential expression under salt stress as compared to normal conditions, and the expression profiles were in accordance with the results obtained by RNA-seq (correlation coefficient = 0.85). Log2 (fold change) values for each DEG are shown in Fig 6.

## Discussion

In this study, we carried out de novo transcriptome sequencing to investigate salt tolerance mechanisms in two jute species under salt stress. In previous studies, some reports showed that the number of DEGs was greater in roots than in leaves underlying salt stress. For example, under salt treatment, 4,884/3,692 up-/downregulated unigenes were discovered in cotton leaf samples; and much more up-/downregulated unigenes (6,303/11,068) were discovered in cotton roots DEGs[25]. while Yong et al. reported that[26] the number of DEGs was slightly greater in the roots (8,665 DEGs) than those in the leaves (7,795 DEGs) in *Brassica napus*. The number of DEGs in different tissues (e.g. roots and leaves) under salt stress might be related to crops. In the current study, much more DEGs common to both species in the roots than in leaves were discovered in jute, more similarly with in cotton; probably because that they were both classified as the Malvaceae[27]. Results of these studies might imply that roots were the main tissue responding to salt stress, especially in Malvaceae crops. In total, 127 DEGs were common to both tissues and both species, and the positive regulation DEGs (107) indwelled in all tissues and species were more than passive regulation DEGs (12) suggesting that the DEGs might play important roles in response to environmental stress in all of tissues and species.

Most of the identified DEGs common to both tissues and both species were directly related to the response to environmental stress. For example, 13 DEGs were enriched in plant hormone signal transduction, and most of these were upregulated; DEGs involved in oxidation-reduction and protein phosphorylation were also highly represented. Some DEGs, played important roles in salinity stress, were discovered. For example, there was evidence supports that HKT participated in the pathways of Na+ entry into the roots subjected to high salinity; and then Na+ was perceived and changed concentration of cytosolic Ca2+ decoded by Ca2+-sensing proteins, such as CIPK. Oscillations of Ca2+ induced by salt stress were sensed by SOS3, a myristoylated Ca2+-binding protein; finally, the SOS1 was phosphorylated and activated through SOS pathway which extruded sodium in cells[28]. In addition, NHXs could compartmentalize Na+ in vacuoles to prevent toxic effects in the cytosol. In the study, numerous DEGs encoding HKT (1, up-regulated), CIPK (27, 17 up-regulated,), SOS1 (2, up-regulated) and NHX (3, up and down-regulated) were found[29]. The DEGs might be play crucial role in Na+ homeostasis. previous studies have shown that TF families such as AP2/EREBP, HB, MYB, NAC, bZIP, HSF, Orphans, FHA, and WRKY are linked to salt stress[30, 31]. In the present study, DTFs expressed in leaves and roots differed; 17 DTF families including HB and HSF were detected in the former, while in the latter, 43 families including MYB, WRKY, and CCAAT were represented. Interestingly, 11 DTFs were common to both tissues in both species that are likely critical for the response to salt stress.

Pathways obviously involved in the salt stress response were identified in the study. The pathways should be important for plants in response to abiotic stress. However, some Pathways were only enriched in roots or leaves in NY and YY, such as TCA cycle and Cutin, suberine and wax biosynthesis. For example, TCA cycle was obviously enriched in root of both species in this study, consistent with previous some studies. Kreuzwieser et al. proposed that hypoxia stress led to the inhibition of the TCA cycle and differential expression of some genes involved in TCA cycle only in roots of poplar trees[32]. And cutin, suberine and wax biosynthesis pathway was only enriched in leaves in NY and YY, in previous studies, the pathway was enriched in leaves[33] or in roots[34], but Lauter et al. reported cutin, suberine and wax biosynthesis term was enriched only in leaves of soybean under iron deficiency stress. suggesting in which tissues cutin, suberine and wax biosynthesis pathway was enriched might be influenced by numerous factors, such as species, stress conditions etc. Cutin, suberine and wax played important roles at plant environment interfaces by serving as a barrier controlling the movements of water and solutes. Which was thus important for the abilities of plants to withstand various abiotic stresses, such as drought and salinity [35]. So, which cutin, suberine and wax biosynthesis pathway was not enriched in root in NY and YY might result in uptaking more ion inside the root tissues from the rhizosphere and finally causes jute susceptibility under salinity stress.

The ABA signaling pathway is central to the salt stress responses in plants[1]. In this study, 20 DEGs were associated with this pathway, including genes encoding PP2C, SnRK2, and ABF (upregulated) and PYL (downregulated). Although PYL and PP2C expression is expected to be positively correlated based on what is known of ABA signaling, our observations are supported by recent studies[36, 37]. The cytokinin signaling pathway includes cytokinin receptors; membrane-bound histidine kinases (e.g., CRE1) with a cytokinin-binding domain; AHP (which transmits signals to the nucleus); and B- and A-type ARR, which are phosphorylated by AHP in the nucleus[38, 39]. Plants lacking or harboring mutant CRE1 show increased tolerance to high salt and drought conditions[40]; furthermore, B- and A-ARR mutations also increase salt tolerance[41], implying that cytokinin signaling negatively regulates the response to salt stress. In the present study, CRE1 and B- and A-ARR were downregulated in roots and leaves of both *Corchorus* species in response to high salinity. However, most AHP genes were upregulated, suggesting that they negatively regulate cytokinin signaling[42] to increase salt tolerance in plants.

CaM and CML are Ca^2+^ sensors that transmit Ca^2+^ signals to downstream effectors under conditions of environmental stress. Ca^2+^-loaded CaM/CML interacts with and regulates a broad spectrum of proteins including TFs, protein kinases and phosphatases, and metabolic enzymes[43]. Overexpression of CaM in *Arabidopsis* as a result of high salinity conferred salt stress tolerance via upregulation of the DNA-binding activity of MYB[44]. Interestingly, 20 DEGs corresponding to CML proteins were detected while MYB was the most highly represented TF family in YY and NY roots. CAMK upregulated in both YY and NY roots may enhance tolerance to salt stress[45] and interact with serine/threonine kinases that activate a Na+/H+ antiporter, resulting in the extrusion of Na+ into soil or xylem for transporting to leaves[46]. CAMK and the serine/threonine protein kinase PP3C may activate Ca^2+^ and MAPK signaling pathways in plants in response to high salt conditions or drought[10]. H^+^-ATPases were highly expressed in roots under salt stress, likely providing the proton-motive force required for maintaining membrane potential[47]. In the roots of both Corchorus species, there were 19 DEGs encoding H+-ATPase subunits under salt stress. This is consistent with previous studies[47] and demonstrates the importance of H^+^-ATPases for salt tolerance in plants.

In conclusion, our results provide a comprehensive resource for investigations into the mechanisms of salt response in plants, and can serve as a basis for breeding salt-tolerant cultivars of *Corchorus* species in the future.

## Supporting information

S1 File Primers for the DEGs and ELF gene. (XLSX)

S2 File All differentially expressed unigenes (DEGs) in NYSR (NY salt-stressed root) vs. NYCR (NY control root); NYSL (NY salt-stressed leaf) vs. NYCL (NY control leaf); YYSR (YY salt-stressed root) vs. YYCR (YY control root); YYSL (YY salt-stressed leaf) vs. YYCL (YY control leaf). (XLSX)

S3 File Common differentially expressed unigenes (DEGs) in roots across both species. (XLS)

S4 File Common differentially expressed unigenes (DEGs) in leavies across both species. (XLS)

S5 File Common differentially expressed unigenes (DEGs) across both tissues and species. (XLS)

S6 File Transcription factors (TFs) identified in the study. (XLSX)

S7 File In the leaves, 32 differentially expressed transcription factors (DTFs) common to the two species. (XLSX)

S8 File In the roots, 196 differentially expressed transcription factors (DTFs) common to the two species. (XLSX)

S9 File Eleven differentially expressed transcription factors (DTFs) common to both tissues and both species. (XLSX)
